# Stability and Change of the Personality Traits Languidity and Flexibility in a Sample of Nurses: A 7–8 Years Follow-Up Study

**DOI:** 10.3389/fpsyg.2021.652569

**Published:** 2021-07-29

**Authors:** Ståle Pallesen, Eirunn Thun, Siri Waage, Øystein Vedaa, Anette Harris, Kjersti Marie Blytt, Puneet Kaur, Bjørn Bjorvatn

**Affiliations:** ^1^Department of Psychosocial Science, University of Bergen, Bergen, Norway; ^2^Norwegian Competence Center for Sleep Disorders, Haukeland University Hospital, Bergen, Norway; ^3^Optentia, The Vaal Triangle Campus of the North-West University, Vanderbijlpark, South Africa; ^4^Department of Global Public Health and Primary Care, University of Bergen, Bergen, Norway; ^5^Department of Health Promotion, Norwegian Institute of Public Health, Bergen, Norway; ^6^Department of Mental Health, Norwegian University of Science and Technology, Trondheim, Norway; ^7^Voss District Psychiatric Hospital, NKS Bjørkeli, Voss, Norway; ^8^Department of Research and Development, St. Olav’s University Hospital, Trondheim, Norway; ^9^Department of Health and Caring Sciences, Western Norway University of Applied Sciences, Bergen, Norway

**Keywords:** change, circadian type inventory-revised, flexibility, languidity, reliability, stability, longitudinal

## Abstract

The traits languidity (tendency to become tired/sleepy upon losing sleep) and flexibility (ability to sleep and work at odd times) have been implicated in shift work tolerance. However, there is a dearth of knowledge about their temporal stability. The aim of the present study was to explore these traits during a long follow-up (FU) period and identify factors related to potential changes in trait scores over time. In all, 1,652 nurses completed the Circadian Type Inventory-revised (CTI-r), which measures languidity and flexibility, at both 2008/2009 (baseline, BL) and again in 2016 (FU). The latent scores of these two constructs at BL, in addition to age, sex, childcare responsibility, marital status, night work status, and insomnia status, were regressed on the corresponding latent scores at follow-up using a structural equation modeling (SEM) approach. Stability was found for both languidity (rho = 0.59) and flexibility (rho = 0.58). Both composite scores declined significantly from baseline (20.62 and 12.48) to follow-up (19.96 and 11.77). Languidity at baseline was positively associated with languidity at follow-up (*β* = 0.89, *p* < 0.009). Undertaking childcare responsibility between baseline and follow-up was inversely related to languidity at follow-up (*β* = −0.09, *p* < 0.05). Starting night work was positively related to languidity at follow-up (*β* = 0.06, *p* < 0.05). Developing insomnia between baseline and follow-up (*β* = 0.15, *p* < 0.05) was positively, whereas remitting from insomnia during the same period was negatively (*β* = −0.11, *p* < 0.01) associated with languidity at follow-up. Flexibility at baseline was positively associated with flexibility at follow-up (*β* = 0.64, *p* < 0.05). Having childcare responsibility at baseline, but not at follow-up was inversely related to flexibility at follow-up (*β* = −0.05 *p* < 0.05). Becoming cohabitant with a partner between baseline and follow-up (*β* = −0.07, *p* < 0.05) was negatively associated with flexibility at follow-up. Starting night work between baseline and follow-up (*β* = 0.17, *p* < 0.01) and reporting night work at both baseline and follow-up (*β* = 0.18, *p* < 0.01) were both positively associated with flexibility at follow-up, whereas stopping working nights was negatively (*β* = −0.09, *p* < 0.05), associated with flexibility at follow-up. The personality traits languidity and flexibility show fairly high stability, albeit the mean scores were significantly reduced during the 7–8 years follow-up period. The results suggest that these personality traits are partly modifiable.

## Introduction

There are large individual differences in the ability to cope with shift and night work. Accordingly, Andlauer et al. (1979) defined shift work tolerance as the ability to adapt to shift work without adverse consequences. Research has found shift work tolerance to be related to demographic, personality, and genetic factors (Saksvik et al., 2011). A pair of characteristics that have received attention in this regard are the personality traits “flexibility” and “languidity” (Di Milia et al., 2005). Languidity concerns difficulties overcoming drowsiness and feelings of lethargy following sleep loss. This trait has been considered to mirror low amplitude circadian rhythms (Di Milia et al., 2005) and correlates significantly with self-reported sleepiness (Roodbandi et al., 2015). The other trait, flexibility, denotes the ability to sleep and work at unusual hours and is assumed to reflect non-stable circadian rhythms (Folkard et al., 1979). Accordingly, flexibility correlates negatively with actigraphy recorded inter-daily stability in activity levels (Boudebesse et al., 2014). The two traits are normally assessed by the Circadian Type Inventory-revised (CTI-r; Di Milia et al., 2005).

Overall, findings from both cross-sectional and longitudinal studies suggest that languidity is inversely related to shift work tolerance, whereas flexibility is positively associated with shift work tolerance (Natvik et al., 2011; Saksvik et al., 2011; Saksvik-Lehouillier et al., 2012, 2013; Storemark et al., 2013; Vedaa et al., 2016; Booker et al., 2018; Hosseinabadi et al., 2019). Although assumed to be fairly stable traits, there is currently lack of evidence regarding their temporal stability. However, in a small sample of bipolar patients (*n* = 19), the 6-month test–retest correlation coefficient for the languidity and flexibility subscales was 0.72 and 0.62, respectively (Boudebesse et al., 2013), suggesting relatively high stability. In addition to the small amount of longitudinal studies involving languidity and flexibility, no study has so far investigated factors that might be related to changes in the scores of these traits over time. Such potential factors might be age and sex, as it has been shown that young age and male sex are associated with higher shift work tolerance (Saksvik et al., 2011). Furthermore, night work and childcare responsibility might also influence how languidity and flexibility are perceived, as studies have shown that childcare responsibility and night work status over time could predict changes in the morningness-eveningness dimension, another self-reported circadian parameter (Vedaa et al., 2013). Insomnia status is further a potential predictor of languidity and flexibility, as the ability to maintain good sleep is regarded as an important aspect of shift work tolerance (Jung and Lee, 2015). In addition, as married individuals report better health than their non-married counterparts (Joung et al., 1994), the influence of marital status on the stability of languidity and flexibility also needs to be investigated.

Against this backdrop, we investigated the stability of the languidity and flexibility traits in a large sample of nurses over a period of 7–8 years. Furthermore, we investigated whether age, sex, marital status, childcare responsibility, night work status, and insomnia status would predict changes in languidity and flexibility over the same time period.

## Materials and Methods

### Sample and Procedure

The present study was based on longitudinal data from SUSSH, an ongoing study exploring work and health status of Norwegian nurses. The first data collection took place in 2008/2009 (baseline, BL), with annual follow-ups (FUs). The eighth data collection took place in 2016 (follow-up), a follow-up time of about 7–8 years. Originally, 6,000 nurses (all members of the Norwegian Nurses Organization) were invited to part take in SUSSH. The nurses were randomly selected from equal strata based on years, since completion of basic nursing education (0–1, 1.1–3, 3.1–6, 6.1–9, and 9.1–12 years). A total of 2,059 nurses participated at baseline (2,059/5,400) amounting to a response rate of 38.1% when removing returns due to wrong addresses (*n* = 600). About 1 year later (2009), 2,741 newly graduated nurses were invited to participate, of which 905 agreed, yielding a response rate of 33.0%. These two groups together formed the baseline cohort of the SUSSH. At follow-up, 1,826 participated which equaled 61.6% of all individuals who participated at baseline. A total of 1,652 nurses had valid scores on all items of the CTI-r at both baseline and follow-up, and hence constituted the analytic sample.

### Instruments

Information about age and sex were provided at baseline. At both baseline and follow-up, the nurses reported whether they lived together with at least one child or not, whether they were married/lived with a partner or not, and whether they had a work schedule that included night work (at least 3 h of work between midnight and 05:00; Stevens et al., 2011).

The Bergen Insomnia Scale (BIS) is a self-reporting measure of insomnia consisting of six items, each scored on an eight-point scale reflecting the number of days per week a specific symptom is experienced (Pallesen et al., 2008). The BIS is constructed based on the inclusion criteria for insomnia found in the 4th edition of the Diagnostic and Statistical Manual for Mental Disorders (DSM-IV; American Psychiatric Association, 1994). The time frame for the symptoms was 1 month at baseline. At follow-up, the time frame was 3 months, in line with the revised insomnia criteria in the 5th edition of the DSM (American Psychiatric Association, 2013). Insomnia was deemed present if the nurse reported at least one of the following problems at least three times per week: problems falling asleep, problems maintaining sleep, early morning awakening, or non-restorative sleep and at least one of the following at least three times per week: sleep dissatisfaction or daytime impairment (Pallesen et al., 2008). The Cronbach alpha of the BIS was 0.83 at baseline and 0.84 at follow-up, respectively.

The CTI-r is an 11-item self-report scale reflecting languidity (six items), which concerns difficulties overcoming drowsiness and feelings of lethargy following reduction in sleep, and flexibility (five items), which denotes the ability to sleep and work at odd times. The responses are rated on a five-point scale ranging from 1 (almost never) to 5 (almost always) in which high scores indicate a tendency toward possessing the trait to a high degree. The CTI-r has been shown to possess high reliability and validity in a working sample (Di Milia et al., 2005). Cronbach alphas for the languidity subscale were 0.69 and 0.71 at baseline and follow-up, respectively, whereas the corresponding values for the flexibility subscale were 0.81 and 0.83.

### Statistics

The analyses were conducted by IBM SPSS version 25, and IBM SPSS AMOS, version 26. In order to compare those with valid answers on the CTI-r at both baseline and follow-up (*n* = 1,652) to those without valid answers on the CTI-r at both time points (*n* = 1,313), chi-square analyses (for nominal variables) and *t*-tests for independent samples (for interval and ratio variables) were used. The groups were compared on the baseline values on age, sex, languidity, flexibility, childcare status, marital status, night work status, and the insomnia score on the BIS. T-tests for paired samples were used in order to investigate changes in the scores on the composite scores of the languidity and flexibility subscales over the 7–8 years period. The measurement model of the CTI-r at baseline was investigated using a confirmatory factor analysis (CFA). The comparative fit index (CFI), and the root mean square error of approximation were used as fit indexes. As a rule of thumb, a model with acceptable fit to the data has CFI > 0.90, and a RMSEA < 0.08, whereas a model with good fit will have CFI > 0.95, and RMSEA < 0.06, respectively (Hu and Bentler, 1999). Measurement invariance between baseline and follow-up was tested in terms of configural invariance (whether the overall factor structure fitted equally at baseline and follow-up) and metric invariance (examining whether the factor loadings were equivalent across the two time points). A common criterion for invariance for nested models is that ΔCFI < 0.01 (Cheung and Rensvold, 2002). In order to investigate the 7–8 years test–retest reliability of the composite score of each subscale, the Spearman rank-order correlation coefficient between the two measurement points was calculated.

**Figure 1:**
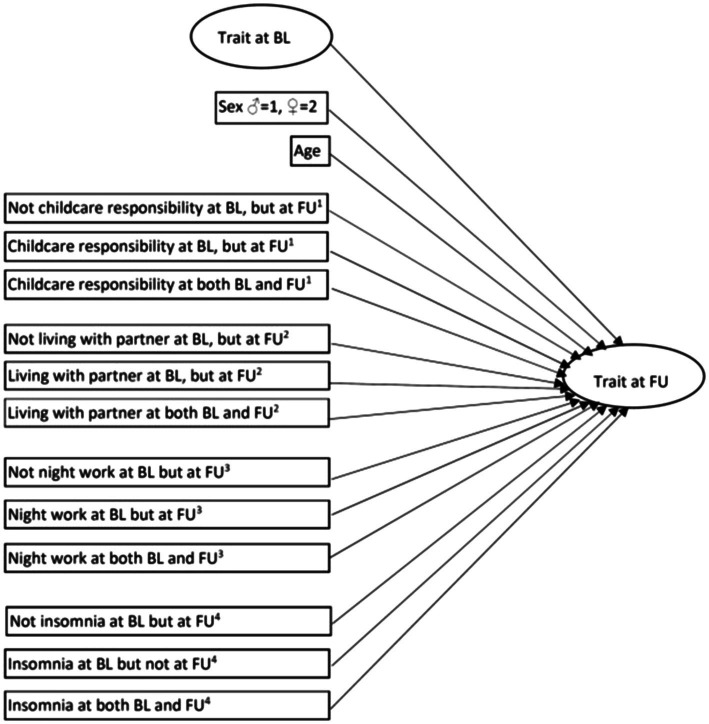
The structural equation model (SEM; BL = baseline, FU = follow-up, ^1^Not having childcare responsibility at neither BL nor FU as reference category, ^2^Not living with partner at neither BL nor FU as reference category, ^3^Not having night work at neither BL nor FU as reference category, and ^4^Not insomnia at neither BL nor FU as reference category).

A structural equation modeling (SEM) approach was used to investigate factors (expressed in terms of standardized regression coefficients) that might predict the two latent personality variables at follow-up. Independent variables were the corresponding latent personality variable at baseline. Other independent variables, represented by manifest variables, were also included in the model: (a) age, (b) sex (♂ = 1, ♀ = 2), (c) childcare responsibility (childcare responsibility at follow-up but not at baseline, childcare responsibility at baseline but not at follow-up, childcare responsibility both at baseline and follow-up) in which not childcare responsibility at neither baseline nor follow-up comprised the reference category, (d) marital status (living with partner at follow-up but not at baseline, living with partner at baseline but not at follow-up, living with partner at both baseline and follow-up) in which neither living with partner at baseline nor at follow-up comprised the reference category, (e) night work (night work at follow-up but not at baseline, night work at baseline but not at follow-up, night work at both baseline and follow-up) in which not night work at neither baseline nor follow-up comprised the reference category, and (f) insomnia status (insomnia at follow-up but not at baseline, insomnia at baseline but not at follow-up, insomnia at both baseline and follow-up) in which not having insomnia at neither baseline nor follow-up comprised the reference category. The analytic model is shown in Figure 1.

## Results

Table 1 presents an overview of the categorical study variables. The mean age at baseline was 31.9 years (SD = 8.4). In Table 2, comparisons between those with and without valid answers on the CTI-r at both baseline and follow-up for age, sex, languidity, flexibility, childcare status, marital status, night work status, and the insomnia score on the BIS are shown. The two groups differed on three of the eight variables. Among those with valid answers on the CTI-r at both baseline and follow-up, fewer had caretaker responsibility for children and were in a relationship compared to those without valid answers on the CTI-r at both time points. In addition, the former group had statistically significant lower score on the BIS compared to the latter group.

**Table 1:** Overview of the categorical study variables for the nurses (*N* = 1,354–1,628) covering the 7–8 years follow-up period.

Variable	Percentage (%)
Sex	
Male	9.1
Female	90.9
Childcare status	
Not living with child at baseline or follow-up	23.9
Living with child at baseline but not at follow-up	6.0
Living with child at follow-up but not at baseline	35.2
Living with child at baseline and follow-up	34.9
Marital status	
Not living with partner at baseline or follow-up	14.3
Living with partner at baseline but not at follow-up	5.6
Living with partner at follow-up but not at baseline	18.1
Living with partner at baseline and follow-up	62.0
Night work status	
Not night work at baseline or follow-up	28.7
Night work at baseline but not at follow-up	27.2
Night work at follow-up but not at baseline	7.1
Night work at baseline and at follow-up	37.0
Insomnia status	
Not insomnia at baseline or at follow-up	34.8
Insomnia at baseline but not at follow-up	18.3
Insomnia at follow-up but not at baseline	15.9
Insomnia at both baseline and at follow-up	30.9

Results are presented as percentages.

**Table 2:** Comparisons of baseline values between those with (*n* = 1,652) and without (*n* = 1,313) valid answers at both baseline and follow-up on the Circadian Type Inventory-revised (CTI-r) on age, sex, languidity, flexibility, child care status, marital status, night work status, and the insomnia score on the Bergen Insomnia Scale (BIS).

Variable	Valid answers at both baseline and follow-up [% or mean (SD)]	Not valid answers at both baseline and follow-up [% or mean (SD)]	*χ*^2^/*df* or *t*/*df*	*p*
Age	31.9 (8.4)	31.7 (8.0)	*t*/2954 = −0.65	0.516
Sex	90.9%♀	90.4%♀	*χ*^2^/1 = 0.17	0.684
Languidity	20.6 (3.7)	20.7 (3.8)	*t*/2853 = 0.35	0.729
Flexibility	12.5 (4.1)	12.4 (4.1)	*t*/2865 = −0.18	0.858
Child care status	Children at home 41.5%	Children at home 46.8%	*χ*^2^/1 = 7.68	0.006
Marital status	In relationship 67.6%	In relationship 73.0%	*χ*^2^/1 = 10.62	0.002
Night work status	Have night work 64.0%	Have night work 64.9%	*χ*^2^/1 = 0.18	0.668
Insomnia score	12.6 (8.1)	13.9 (8.4)	*t*/2907 = 4.37	<0.001

**Figure 2:**
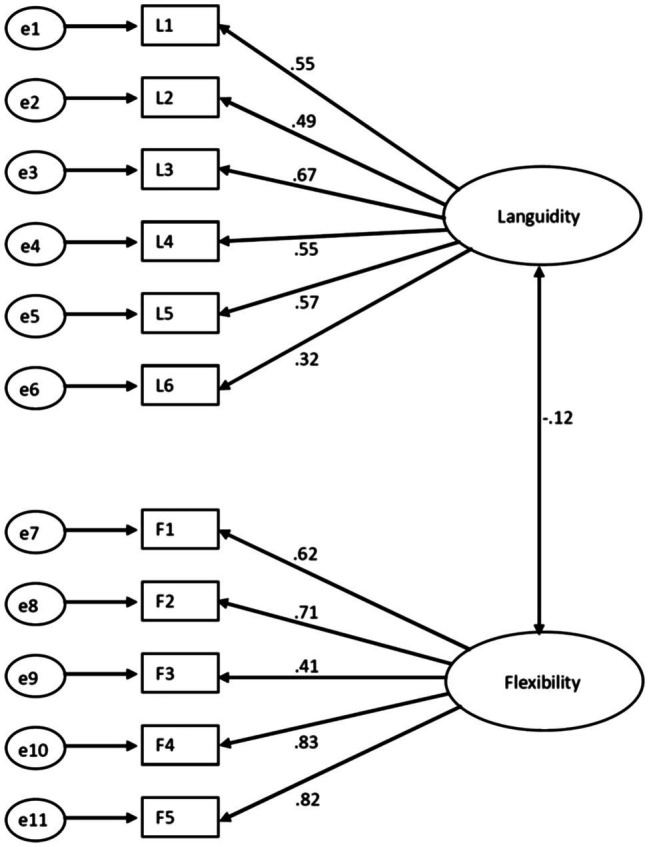
The measurement model at baseline showing the standardized regression coefficients and the correlation coefficient between the two latent variables.

The measurement model at baseline, *χ*^2^ (*df* = 43, *n* = 1,652) = 343.6, had acceptable fit with the data as the CFI was 0.932 and the RMSEA was 0.065 (90% CI = 0.059, 0.072). The factor structure with its standardized regression coefficients is shown in Figure 2. The standardized regression coefficients varied from 0.32 to 0.67 for languidity and 0.41 to 0.83 for flexibility. In terms of invariance between the two time points, the model showed configural invariance [*χ*^2^ (*df* = 86, *n* = 3,304) = 665.8, CFI = 0.938, RMSEA = 0.045 (90% CI = 0.042, 0.046)]. The model also showed metric invariance (Δ*χ*^2^ = 12.g, *df* = 9, *p* > 0.05, ΔCFI = 0.000, ΔRSMEA = 0.002) between baseline and follow-up.

The composite languidity score was reduced from 20.62 (SD = 3.68) to 19.96 (SD = 3.88) from baseline to follow-up (*t* = 8.03, *df* = 1,651, *p* < 0.001). The composite flexibility score was reduced from 12.48 (SD = 4.07) to 11.77 (SD = 4.19) from baseline to follow-up (*t* = 7.68, *df* = 1,651, *p* < 0.001). The test–retest (baseline to follow-up) correlation coefficient (Spearman’s rho) for the languidity and the flexibility subscale were 0.59 and 0.58, respectively.

Table 3 presents the SEM-results for languidity at follow-up as the latent dependent variable.

**Table 3:** Regression analysis summary for languidity at BL, sex, age, childcare status, marital status, night work status, and insomnia status predicting scores on languidity at follow-up (FU; *N* = 1,286).

Variable	*β*	95% CI for *β*	*p*
Languidity at BL	0.89	0.814, 0.945	0.009
Sex (♂ = 1, ♀ = 2)	−0.02	−0.077, 0.035	0.460
Age	−0.01	−0.082, 0.047	0.587
Child care status^a^			
Not childcare responsibility at BL, but at FU	−0.09	−0.166, −0.006	0.035
Childcare responsibility at BL, but not at FU	−0.02	−0.082, 0.039	0.632
Childcare responsibility at both and FU	−0.04	−0.098, 0.052	0.472
Marital status^b^			
Not living with partner at BL but at FU	0.06	−0.014, 0.131	0.104
Living with partner at BL but not at FU	−0.01	−0.065, 0.055	0.788
Living with partner at both BL and FU	−0.00	−0.097, 071	0.961
Night work status^c^			
Not night work at BL but at FU	0.06	0.006, 0.123	0.043
Night work at BL but not at FU	−0.04	−0.105, 0.032	0.256
Night work at both BL and FU	−0.02	−0.084, 0.040	0.506
Insomnia status^d^			
Not insomnia at BL but at FU	0.15	0.076, 0.206	0.013
Insomnia at BL but not at FU	−0.11	−0.179, −0.056	0.005
Insomnia at both BL and FU	0.02	−0.047, 0.099	0.464

*β*, standardized regression coefficient, *R*^2^ = 0.826, CMIN/DF = 5.54, CFI = 0.89, RMSEA = 0.06 (90% CI = 0.056, 0.063).

a Not having childcare responsibility at neither BL nor FU as reference category.

b Not living with partner at neither BL nor FU as reference category.

c Not having night work at neither BL nor FU as reference category.

d Not insomnia at neither BL nor FU as reference category.

Statistically significant predictors were languidity at baseline (*β* = 0.89, *p* < 0.01), not having childcare responsibility at baseline, but having this at follow-up (*β* = −0.09, *p* < 0.05), starting working nights in the follow-up period (*β* = 0.06, *p* < 0.05), having insomnia at follow-up but not at baseline (*β* = 0.15, *p* < 0.001), and remitting from insomnia during the follow-up period (*β* = −0.11, *p* < 0.001). The *R*^2^ was 0.826.

Table 4 shows the SEM-results for flexibility at follow-up as the latent dependent variable.

**Table 4:** Regression analysis summary for flexibility at BL, sex, age, childcare status, marital status, night work status, and insomnia status predicting scores on flexibility at follow-up (FU; *N* = 1,286).

Variable	*β*	95% CI for *β*	*p*
Flexibility at BL	0.64	0.592, 0.684	0.018
Sex (♂ = 1, ♀ = 2)	−0.03	−0.087, 0.011	0.111
Age	0.03	−0.033, 0.085	0.311
Child care status^a^			
Not childcare responsibility at BL, but at FU	−0.01	−0.088, 0.074	0.839
Childcare responsibility at BL, but not at FU	−0.05	−0.098, −0.006	0.024
Childcare responsibility at both and FU	0.00	−0.071, 0.071	0.909
Marital status^b^			
Not living with partner at BL but at FU	−0.07	−0.158, −0.017	0.020
Living with partner at BL but not at FU	0.01	−0.062, 0.066	0.953
Living with partner at both BL and FU	−0.00	−0.082, 0.066	0.861
Night work status^c^			
Not night work at BL but at FU	0.17	0.113, 0.250	0.010
Night work at BL but not at FU	−0.09	−0.124, −0.040	0.016
Night work at both BL and FU	0.18	0.129, 0.236	0.006
Insomnia status^d^			
Not insomnia at BL but at FU	−0.01	−0.055, 032	0.576
Insomnia at BL but not at FU	0.03	−0.088, 114	0.121
Insomnia at both BL and FU	0.02	−0.028, 082	0.336

*β*, standardized regression coefficient, *R*^2^ = 0.539, CMIN/DF = 4.68, CFI = 0.95, RMSEA = 0.05 (90% CI = 0.049, 0.058).

a Not having childcare responsibility at neither BL nor FU as reference category.

b Not living with partner at neither BL nor FU as reference category.

c Not having night work at neither BL nor FU as reference category.

d Not insomnia at neither BL nor FU as reference category.

Statistically significant predictors were flexibility at baseline (*β* = 0.64, *p* < 0.05), having childcare responsibility at baseline but not at follow-up (*β* = −0.05, *p* < 0.05), not living with partner at baseline but living with partner at follow-up (*β* = −0.07, *p* < 0.05), not night work at baseline but night work at follow-up (*β* = 0.17, *p* < 0.01), night work at baseline but not at follow-up (*β* = −0.09, *p* < 0.05), and night work at both baseline and follow-up (*β* = 0.18, *p* < 0.01). The *R*^2^ was 0.539.

## Discussion

Although the composite scores of languidity and flexibility remained fairly stable over the 7–8 years follow-up period, a significant reduction in the composite scores was observed for both traits.

The SEM-analysis showed that languidity at baseline had a strong positive relationship (*β* = 0.89) with languidity at follow-up. Not having childcare responsibility at baseline, but having childcare responsibility at follow-up was inversely related to languidity at follow-up, compared to not having childcare responsibility at neither baseline nor follow-up. Starting night work between baseline and follow-up was positively related to languidity at follow-up compared to the contrast (not night work at neither baseline nor follow-up). Developing insomnia between baseline and follow-up were positively associated with languidity at follow-up, whereas remitting from insomnia during the same period was inversely related to languidity at follow-up compared to the contrast (not having insomnia at neither baseline nor follow-up).

Flexibility at baseline had a strong and positive relationship with (*β* = 0.64) with flexibility at follow-up. The results from the SEM-analysis showed that having childcare responsibility at baseline but not at follow-up was negatively associated with flexibility at follow-up, compared to the contrast not having childcare responsibility at neither baseline and nor follow-up. Further, the results showed that scores were lower for those who started living with a partner between baseline and follow-up compared to those not living with a partner at neither baseline nor follow-up. Furthermore, participants who stopped night work between baseline and follow-up had lower scores on flexibility at follow-up than those who either had night work at both baseline and follow-up. Those reporting night work at follow-up had higher scores on flexibility at follow-up than participants not having night work neither at baseline nor follow-up.

A reduction in the composite languidity score over the follow-up period was found. This might be attributed to decreased amplitude in core body temperature as a function of age (Richardson et al., 1982) and is consistent with epidemiological studies showing an inverse relationship between self-reported sleepiness and age (Pallesen et al., 2007). Also, the composite flexibility score was reduced during the follow-up period. This finding is in line with the majority of studies showing reduced ability to work nights with advancing age (Seo et al., 2000; Chan, 2009). The mechanism behind this is not evident, but might reflect that older subjects are more sensitive to circadian effects on performance than younger subjects (Reid and Dawson, 2001), advancement of the circadian rhythms with age (Van Someren, 2000) and general deterioration of health as subjects become older (Ritonja et al., 2019).

Those not having childcare responsibility at baseline but who reported this at follow-up had a larger decrease in languidity compared to those not having childcare responsibility at neither baseline nor follow-up. One possible explanation to this finding is that those who do not become parents may have less experience in coping with sleep loss, as sleep loss is common among those who become parents (Richter et al., 2019). Starting night work in the follow-up period was positively associated with languidity at follow-up, compared to the contrast not working nights at neither baseline nor follow-up. This is line with studies showing that night work is associated with increased fatigue (Härmä et al., 2019). In addition, developing insomnia between baseline and follow-up were positively related to languidity at follow-up, whereas remitting from insomnia in the same period was inversely related to languidity at follow-up, compared to those who did not have insomnia at either time points. This finding seems conceivably as languidity overlaps with symptoms of daytime consequences of insomnia (American Academy of Sleep Medicine, 2014).

Stopping having childcare responsibility in the follow-up period was inversely related to flexibility at baseline, compared to the contrast, not having childcare responsibility at either time points. Having childcare responsibility typically implies that timing of sleep and wakefulness may be less predictable and self-chosen; hence, timing of sleep and wakefulness may become more rigid when parenting responsibilities cease. Becoming cohabitant with a partner between baseline and follow-up was associated with a relatively larger reduction in flexibility over time compared to the reference category (not living with a partner at neither baseline nor follow-up). According to Sbarra and Hazan (2008) romantic bonds may constitute a form of co-regulation, in which individuals reciprocally maintain physiological functioning in each other. Hence, taking such perspectives into consideration, it might be reasonable to expect that subjects experience stronger entrained rhythms when engaging in relationships, resulting in a reduction of the flexibility trait. The final significant predictor was night work, showing that participants who stopped night work between baseline and follow-up had a steeper reduction in flexibility than those not having night work at neither baseline nor follow-up. Further, those starting with night work between baseline and follow-up and those who reported night work at both baseline and follow-up had relatively smaller reduction in flexibility over time than those working nights at neither baseline nor follow-up. Several studies have shown that night work causes changes in the circadian rhythm (Roach et al., 2001; Vedaa et al., 2013), which presumably would make it easier to fall asleep at odd times of the day. Hence, night work seems to be an external factor which seems to promote flexibility.

### Limitations and Strengths

In terms of limitations, it should be noted that the initial response rate at baseline was mediocre, although commonly regarded as acceptable in these kinds of studies (Baruch and Holtom, 2008). In addition, the sample comprised nurses only, with a large female preponderance, which may put restrictions on the generalizability of the findings. Also, those who did not complete the CTI-r at follow-up were at baseline more often in a relationship, had more often caretaker responsibility for children and had higher scores on the BIS than those with valid answers at both time points. Hence, interpersonal responsibilities and insomnia symptoms were related to attrition, which should be considered when interpreting the findings. Insomnia at baseline was defined based on a 1 month time frame, whereas the time frame at follow-up was 3 months, adhering to diagnostic changes that occurred during the follow-up period (American Academy of Sleep Medicine, 2014), but we have no reason to believe that this had a major influence on the results. Due to missing data, the results from the SEM-analyses were based on somewhat fewer respondents (*n* = 1,286) than the full analytic sample.

Some strengths of the paper also deserve mention. The present paper is the largest longitudinal study to date regarding the languidity-flexibility pair of personality traits. In addition, a follow-up of 7–8 years is the longest follow-up period regarding assessment of these traits reported in the literature. The study was based on a large and homogeneous sample of nurses which limits the influence from possible confounding variables. Another notably strength was the use of validated scales for circadian type (languidity and flexibility) and insomnia. Moreover, the participants were not told that the study had focus on languidity and flexibility, thus reducing the risk of selection bias.

## Conclusion

The personality traits languidity and flexibility were relatively stable over time, although reductions in the scores of both traits over the 7–8 years follow-up period were found. Undertaking childcare responsibility between baseline and follow-up accentuated the reduction in languidity over time.

Starting night work in the follow-up period was positively associated with languidity at follow-up.

Developing insomnia was associated with a more adverse development of the languidity trait, whereas those remitting from insomnia showed an opposite trend. Ceasing to have childcare responsibility and starting to live with a partner and stopping night work between baseline and follow-up were all associated with a steeper reduction in flexibility over time, compared to not having childcare responsibility at neither baseline nor follow-up, not having a partner at neither baseline nor follow-up and not having night work at neither baseline nor follow-up, respectively. Starting night work between baseline and follow-up and having night work at both baseline and follow-up were both associated with a better ability to uphold flexibility scores over time, whereas those stopping night work in the same period showed the opposite trend, compared to those not having night work at neither baseline nor follow-up. Overall, it is concluded that despite being fairly stable personality traits, both languidity and flexibility are to a certain degree modifiable over time.

## Data Availability Statement

The raw data supporting the conclusions of this article will be made available by the authors, without undue reservation.

## Ethics Statement

The study procedures were carried out in accordance with the Declaration of Helsinki and the Norwegian Health Research Act. The study was approved by the Regional Committee for Medical and Health Research Ethics (REC West; no. 088.88) and the Norwegian Data Inspectorate (08/01235/IUR). All participants signed an informed consent form before being included in the study. The patients/participants provided their written informed consent to participate in this study.

## Author Contributions

SP, SW, and BB designed the SUSSH-study. SP, ET, SW, and BB collected the data. SP and PK conducted the analysis. SP drafted the first version of the manuscript. All authors contributed to the interpretation of data, revised the work critically for important intellectual content, approved the final version to be published, and agreed to be accountable for all aspects of the work in ensuring that questions related to the accuracy or integrity of any part of the work are appropriately investigated and resolved.

## Conflict of Interest

The authors declare that the research was conducted in the absence of any commercial or financial relationships that could be construed as a potential conflict of interest.

## Publisher’s Note

All claims expressed in this article are solely those of the authors and do not necessarily represent those of their affiliated organizations, or those of the publisher, the editors and the reviewers. Any product that may be evaluated in this article, or claim that may be made by its manufacturer, is not guaranteed or endorsed by the publisher.
